# Investigation of *Giardia intestinalis* Genotypes among the Food Handlers of Qazvin, Iran

**Published:** 2019

**Authors:** Mojtaba SHAHNAZI, Farzaneh NAGHIZADEH, Elham HAJIALILO, Safar Ali ALIZADEH, Mehrzad SARAEI, Mahmood ALIPOUR

**Affiliations:** 1. Cellular and Molecular Research Center, Research Institute for Prevention of Non-Communicable Disease, Qazvin University of Medical Sciences, Qazvin, Iran; 2. Department of Parasitology and Mycology, Qazvin University of Medical Sciences, Qazvin, Iran; 3. Student Research Committee, Qazvin University of Medical Sciences, Qazvin, Iran; 4. Medical Microbiology Research Center, Qazvin University of Medical Sciences, Qazvin, Iran; 5. Department of Microbiology, Qazvin University of Medical Sciences, Qazvin, Iran; 6. Department of Social Medicine, Qazvin University of Medical Sciences, Qazvin, Iran

**Keywords:** *Giardia*, Food handlers, Glutamate dehydrogenase gene, Iran

## Abstract

**Background:**

We aimed to investigate the genotypes of *Giardia intestinalis* among the food handlers in Qazvin, Iran.

**Methods:**

Overall, 1530 stool specimens were collected from the food handlers who visited Shahid Bolandian Health Center, Qazvin, Iran during 2016. Specimens were evaluated by microscopic and concentration methods. Twenty specimens with appropriate number of giardia cysts were selected followed by DNA extraction**.** Determination of giardia genotypes was achieved through PCR and sequencing the glutamate dehydrogenase gene. The phylogenetic tree was drawn using the MEGA7 software. Finally, the data were analyzed statistically with a *P*-value<0.05 was considered as significant.

**Results:**

Twenty stool samples (1.3%) were positive for *Giardia* cyst. All positive specimens were obtained from male participants with abdominal cramp being their most common symptoms. The mean age for infected individuals was 32 yr. Molecular characterization was successfully performed for 17 isolates and two genotypes A (AII, 65%) and B (BIII, 35%) were identified.

**Conclusion:**

The most prevalent giardia genotypes among the food handlers in Qazvin were A (AII) and B (BIII) genotypes with A (AII) genotype as the dominant one in the region. Considering the direct association between the food handlers and public health as well as the impact of geographical and host conditions on dispersion and pathogenicity of various genotypes and their zoonotic aspects, further investigations are necessary.

## Introduction

G*iardia intestinalis* is an intestinal flagellated protozoon infecting a wide range of vertebrates including the human and domestic and wild animals (1, 2). Giardiasis is considered as a health concern within both the developed and developing countries, reflecting a prevalence rate of 2%–5% in a wide range of developed countries and 20%–30% in developing countries (3, 4).

People with giardiasis in endemic areas are often asymptomatic. Adult individuals are usually the healthy carriers of the parasite whereas children more frequently manifest the signs and symptoms of the disease and giardiasis in developing countries is regarded as a widespread infection of childhood age (5, 6). Transmission of *G. intestinalis* to humans occurs directly or indirectly via accidental ingestion of cysts through contaminated drinking water and foods (7). The availability of adequate sanitation and health care surveillance systems for drinking water and foods, plus the role of food handlers, as the final supplier of food products are of prime importance. Failure of this group of the society in implementing the basic principles of public and personal health and hygiene can lead to transmission of most important pathogenic agents including giardia to humans (8, 9). Several reports from Iran indicate the presence of high prevalence rate of giardiasis in food handlers compared to other intestinal parasites (9–11).

Currently, different genotypes (A-H) have been identified for *G. intestinalis*. Among these genotypes, A and B assemblages have the potential to infect human and other mammals whereas other genetic groupings (C-H) often show host specificity for animals. The human isolates are located in two major genotype groups marked as A and B in which the subgroups Al, All, Blll, and BlV are mostly related to human isolated (12, 13). Several molecular studies in Iran confirm the presence of A and B genotype assemblages as well as Al, All, Blll, and BlV sub-groupings in different areas of the country (14–17).

The molecular study in food handlers infection with giardia was reported in central Iran, so the research was detected AII, BIII, BIV genotypes and mix infection among specimens (18). Considering the relative abundance of giardia in Iran, the significant role of food handlers in public health, and lack of information on giardia and its genotypes in the country, the present research was designed to identify different genotypes of *giardia* among the food handlers of Qazvin Province to provide the health care system with some preliminary information necessary for planning to treat, control, and prevent giardiasis.

## Materials and Methods

### Specimen collection & microscopic examination

This cross-sectional descriptive study performed on a study population consisting of food handlers of Qazvin province (36°15′N, 50°0′E) located in the northern margin of central Iran.

The procedure of this project was approved by the Research Ethics Committee of Qazvin University of Medical Sciences (IR. QUMS. REC. 1396. 293).

Overall, 1530 stool samples were collected from the individuals somehow involved in preparation, distribution, and marketing of food products that visited Shahid Bolandian Health Center for periodic health examination. Specimen collection was carried out from Mar to Aug 2016. The stool specimens were transferred to the Department of Parasitology and Mycology at Qazvin Medical School affiliated to Qazvin University of Medical Sciences, Qazvin, Iran. All fecal samples were examined for the presence of giardia cysts and trophozoites using direct microscopy, formalin-ether acetate, and trichrome staining methods (19, 20).

### Cysts collection and concentration

At this stage, 20 fecal specimens with appropriate number of giardia cysts (an average of 15 cysts per 10 microscopic fields) were selected for concentration assay. The samples were concentrated using sucrose concentration gradient and kept at –20 °C until use (14).

### DNA extraction

The concentrated samples of giardia cysts were washed with dH_2_O 3 times followed by mechanical disruption of giardia cyst wall using glass beads (diameter: 0.45–0.52 mm) and freeze-thawing technique. Later, proteinase K (10 mg/ml) and SDS (1%) were added to the solution and the mixture was incubated at 55 °C for a minimum of 4 h. Finally, DNA extraction was performed using a commercially available specific kit for extracting DNA from stool (QIAamp DNA stool Mini Kit; QIAgen Company) and the samples were stored at –20 °C until the time for PCR assay (15).

### PCR assay

Detection of giardia genotypes was achieved by amplification of glutamate dehydrogenase (GDH) gene using PCR technique. Amplification of the 458-bp DNA segment was conducted using specific primers (BioEdit software) as follows: forward primer: 5' GCTTCCGTATTCAGTACAAC 3' and reverse primer: 5' CTTGCACATCTCCTCCAG 3 '. PCR reaction was performed in a total volume of 20 μl containing ready-made mixture of Ampliqone (Taq DNA Polymerase Master Mix RED, Denmark), Taq Master Mix, template DNA, 0.1 μM of each primer and distilled water. The temperature conditions of PCR reaction in thermal cycler machine were 95 °C for 5 min; 35 cycles of 95 °C for 20 sec, annealing step performed at 55 °C for 20 sec and extension was 75 °C for 30 sec; final extension step was at 72 °C for 5 min. Eventually, the PCR product was electrophoresed on 1% agarose gel and further stained with ethidium bromide.

### Sequencing & phylogenetic analysis

The PCR product of each sample was later purified and sequenced by ABI 3130X sequencer. The sequence of PCR products was analyzed by Chromas (http://technelysium.com.au/wp/chromas/) and the genotypes of giardia identified in our experiments were compared with known genotypes deposited in the database of GenBank (https://www.ncbi.nlm.nih.gov/genbank/) using BLAST. Finally, the genotype of each isolate was determined and then the phylogenetic tree or evolutionary diagram of isolates was drawn according to the Tamura 3-parameter model and MEGA7 software to examine and identify the location of *G. intestinalis* genotypes. Bootstrap analysis based on 1000 replications was applied to draw the phylogenetic tree while *Giardia muris* was regarded as outgroup.

### Statistical analysis

Data were analyzed by SPSS ver.19 (Chicago, IL, USA) using the chi-square test while a *P-*value <0.05 was considered as significant.

## Results

The present study examined 1530 fecal specimens collected from the food handlers of Qazvin among those 20 isolates (1.3%) were positive for *G. intestinalis*. The mean age of people infected with giardia was 32 with the lowest and highest age of 18 and 63 yr, respectively. The highest level of infection was observed in age groups less than 39 yr old and the lowest in those more than 50 yr old. The most prevalent clinical sign among the participants was abdominal cramp 50% (10/ 20) while the less commonly observed sign was weight loss 5% (1/20) (Table 1).

**Table 1:** Gastrointestinal signs distribution of *Giardia intestinalis* isolates in age groups of food handlers in Qazvin, Iran

***Age groups (yr)***	***Gastrointestinal***			***Signs***
	Weight Loss	Diarrhea	Abdominal	No symptoms
	(%)	(%)	pain (%)	(%)
30 <	1 (100)	2 (66)	4 (40)	1 (17)
39–30	0 (0)	0 (0)	4 (40)	4 (66)
49–40	0 (0)	1 (34)	1 (10)	0 (0)
59–50	0 (0)	0 (0)	1 (10)	0 (0)
60 >	0 (0)	0 (0)	0 (0)	1 (17)

**Fig. 1:**
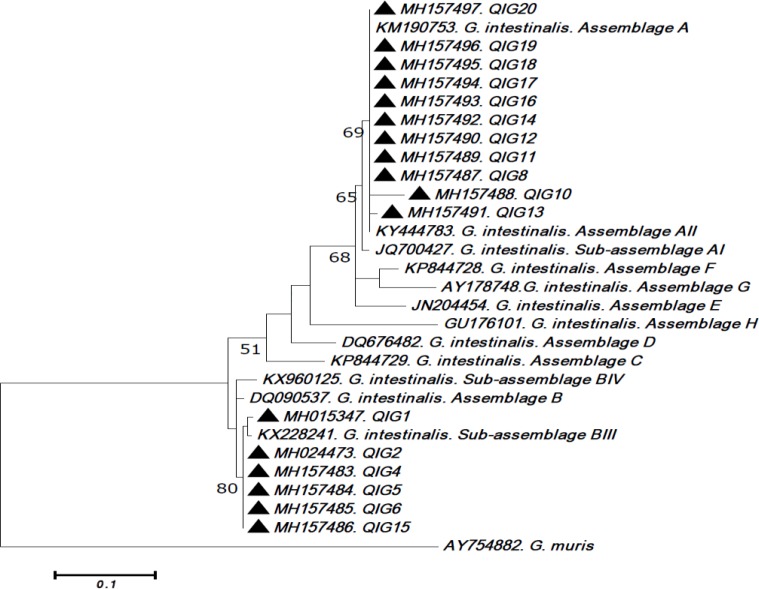
Phylogenetic tree of *G. intestinalis* isolates among the food handlers of from Qazvin, Iran. The specimens were clarified in the present study (▴). The reference sequences retrieved from GenBank based on gdh nucleotide sequences, and then it was constructed by using the Tamura 3-parameter model in MEGA7 software version

PCR reaction produced positive results for 17 isolates out of 20. The results obtained for GDH gene sequence and phylogenetic analysis of sequence of isolates indicated that all isolates carried the assemblage A and B. Obviously, 11 specimens 65% belonged to the sub-assemblage AII and the other 6 (35%) to sub-assemblage BIII. The sequences found for genotypes were recorded in GenBank database (Table 2, Fig.1). In all individuals who suffered giardiasis with or without gastrointestinal signs, the genotype AII was more prevalent compared to BIII genotype. There was no significant correlation between the parasite genotypes and the clinical signs, age, and occupation. Distribution of different genotypes and occupation shown in Table 3.

**Table 2:** *Giardia intestinalis* assemblage among the food handlers of Qazvin, Iran

***Number***	***Code***	***Assemblage***	***Accession no.***
1	QIG1	B	MH015347
2	QIG2	B	MH024473
3	QIG4	B	MH157483
4	QIG5	B	MH157484
5	QIG6	B	MH157485
6	QIG8	A	MH157487
7	QIG10	A	MH157488
8	QIG11	A	MH157489
9	QIG12	A	MH157490
10	QIG13	A	MH157491
11	QIG14	A	MH157492
12	QIG15	B	MH157486
13	QIG16	A	MH157493
14	QIG17	A	MH157494
15	QIG18	A	MH157495
16	QIG19	A	MH157496
17	QIG20	A	MH157497

QIG: Qazvin Isolate Giardia

**Table 3:** Frequency of *Giardia intestinalis* genotypes among the food handlers in Qazvin, according to the profession

***Variable***	***Genotypes***	
Profession	**AII (%)**	**BIII (%)**
Company of foods production	4 (33.4)	1 (16.7)
Kindergartens	1 (8.3)	0 (0)
Restaurant	1 (8.3)	2 (33.2)
Confectionary	0 (0)	1 (16.7)
Bakery	1 (8.3)	0 (0)
Fast Foods Center	2 (16.7)	1 (16.7)
Super Market	2 (16.7)	1 (16.7)

## Discussion

Of 1530 stool samples, 20 cases (1.3%) were positive for *G. lamblia*. The sequence of 11 samples was identical with genotype A (AII) and 6 samples showed a sequence similar to genotype B (BIII). Consistent with this study, some published reports from different geographical regions also have described the isolation of *G. intestinalis* genotypes A and B with genotype A as the dominant genotype. Likewise, in these studies, comparable with our study, the reported genotypes were associated with subtypes AII and BIII, respectively (21, 22). Simultaneous presence of genotypes A and B is reported (23, 24). Furthermore, different genotypes including AII, BIII, and BIV were reported among human isolates of *G. intestinalis* (17, 25).

The studies in Iran indicated the presence of genotypes A and B alone or in combination among the *G. intestinalis* parasite isolated from samples (14, 19). In addition to the presence of genotypes, AII and BIII in their *G. intestinalis* isolates, the isolation of genotype AI was reported from their specimens which highlights the importance of the role of geographical regions in the dissemination of various genotypes of giardia and their regional dominance (15). In other studies, the authors reported the dominance of B (BIII) genotype over A (AII) genotype as well as the isolation of BIV genotype (16, 17). The data of the present study and those reported by aforementioned researchers, in addition to emphasizing the role of environmental factors in spreading diverse *G. intestinalis genotypes*, can be applied to different giardiasis control programs.

The isolation of *G. intestinalis* genotypes A and B from different animals is also reported in several studies (23, 24, 26). Thus, the question on potential of giardia to be considered as a zoonotic parasite remains unclear until further molecular investigations in both human and animals could decisively provide the appropriate information for this hypothesis.

In this study, the prevalence rate of different genotypes of *G. lamblia* in food handlers showed no significant difference according to age groups, occupation, and gastrointestinal symptoms. Nevertheless, the infection rate with genotype AII was higher, compared to BIII, and in all age groups except the group aged 30–39 yr. The individuals in this age group (30–39) demonstrated equal infection rate with AII or BIII genotypes. This lack of significant difference could also be related to the low number of specimens tested. Consistent with our study, the dominant genotype at different ages was the genotype (A) reported from Kerman, Iran. In contrast with the present study, a significant correlation was reported between age and the type of genotype from Argentina and dominant type was genotype B. This discrepancy could be due to the existence of more changes in genotypes within the genotype B group which needs further in-depth studies (15, 27).

In the current study, despite lack of significant correlation between the *G. lamblia* genotypes in infected food handlers, infection with giardia in all occupational groups except the Kababi café & restaurant group was due to genotype AII, whereas in those working in Kababi café & restaurants, infection was produced by both genotypes and that the infection with genotype BIII was higher than genotype AII. Considering the direct association of this group of the society with public health, any type of study encompassing this topic, could be beneficial and of great importance.

In this study, there was no significant correlation between the different genotypes of *G. lamblia* (AII and BIII) and gastrointestinal signs and symptoms however those infected with giardia and showing gastrointestinal symptoms were mostly infected with genotype AII rather than genotype BIII. Moreover, the individuals with giardiasis, but without gastrointestinal signs and symptoms, were exclusively infected with A (AII) genotype. Numerous studies in agreement with our findings found no significant correlation between the genotypes responsible for giardiasis and gastrointestinal signs and symptoms (19, 22, 28). On the contrary, several studies reported from different parts of Iran including Kerman, Tabriz, Shiraz, and Shahrekord (16, 17, 29) and other parts of the world have described a significant correlation between the type of *G. lamblia* genotype and the presence of gastrointestinal symptoms (23, 30). In this respect, several studies from Iran and other geographical regions from Kerman, East Azerbaijan, Holland, Ethiopia demonstrated that the signs of persistent severe diarrhea were due to genotype B whereas those of mild and intermittent diarrhea was caused by genotype B (15, 19, 23, 28). By contrast, in Shiraz, Spain, Turkey the results were quite the opposite i.e. the occurrence of severe diarrhea was associated with genotype A (17, 30, 31). Furthermore, in some of the reports mentioned above the presence of abdominal cramp was mostly linked to genotype A (AII) (17, 32).

Considering the results of the present study and those of other studies, a correlation between the appearance of gastrointestinal symptoms and different genotypes of giardia could confirm the contribution of host factors such as immune system, virulence of different genotypes of giardia, and other unknown factors which need further investigations.

## Conclusion

Different specimens’ positive for *G. intestinalis* were associated with the presence of genotypes A (AII) and B (BIII) isolates with type A as the dominant genotype of the region. Considering the studies reported from Iran and other countries, factors such as geographical conditions, target genes amplification, and host conditions could affect the dissemination and pathogenicity of different genotypes as well as the zoonotic aspects of *G. lamblia* parasite which require more detailed future studies.
